# Women’s experience with home-based self-sampling for human papillomavirus testing

**DOI:** 10.1186/s12885-015-1804-x

**Published:** 2015-11-04

**Authors:** Farhana Sultana, Robyn Mullins, Dallas R. English, Julie A. Simpson, Kelly T. Drennan, Stella Heley, C. David Wrede, Julia M. L. Brotherton, Marion Saville, Dorota M. Gertig

**Affiliations:** Centre for Epidemiology and Biostatistics, Melbourne School of Population and Global Health, University of Melbourne, Melbourne, Australia; Cancer Council Victoria, 615 St Kilda Rd, Melbourne, Vic 3004 Australia; Victorian Cervical Cytology Registry, PO Box 161, Carlton South, Vic 3053 Australia; VCS Inc, 265 Faraday Street, Carlton, Vic 3053 Australia; VCS Pathology, 265 Faraday Street, Carlton, Vic 3053 Australia; Royal Women’s Hospital, Locked Bag 300, Cnr Flemington Road and Grattan Street, Parkville, VIC 3052 Australia; Department of Obstetrics & Gyneacology, University of Melbourne, Melbourne, Australia; National HPV Vaccination Program Register, Victorian Cytology Service, PO Box 310, East Melbourne, Vic 3002 Australia

**Keywords:** Barriers, Cervical screening, Self-sampling, HPV DNA testing, Non-attendees, Never-screened, Under-screened

## Abstract

**Background:**

Increasing cervical screening coverage by reaching inadequately screened groups is essential for improving the effectiveness of cervical screening programs. Offering HPV self-sampling to women who are never or under-screened can improve screening participation, however participation varies widely between settings. Information on women’s experience with self-sampling and preferences for future self-sampling screening is essential for programs to optimize participation.

**Methods:**

The survey was conducted as part of a larger trial (“iPap”) investigating the effect of HPV self-sampling on participation of never and under-screened women in Victoria, Australia. Questionnaires were mailed to a) most women who participated in the self-sampling to document their experience with and preference for self-sampling in future, and b) a sample of the women who did not participate asking reasons for non-participation and suggestions for enabling participation. Reasons for not having a previous Pap test were also explored.

**Results:**

About half the women who collected a self sample for the iPap trial returned the subsequent questionnaire (746/1521). Common reasons for not having cervical screening were that having Pap test performed by a doctor was embarrassing (18 %), not having the time (14 %), or that a Pap test was painful and uncomfortable (11 %). Most (94 %) found the home-based self-sampling less embarrassing, less uncomfortable (90 %) and more convenient (98 %) compared with their last Pap test experience (if they had one); however, many were unsure about the test accuracy (57 %). Women who self-sampled thought the instructions were clear (98 %), it was easy to use the swab (95 %), and were generally confident that they did the test correctly (81 %). Most preferred to take the self-sample at home in the future (88 %) because it was simple and did not require a doctor’s appointment. Few women (126/1946, 7 %) who did not return a self-sample in the iPap trial returned the questionnaire. Their main reason for not screening was having had a hysterectomy.

**Conclusions:**

Home-based self-sampling can overcome emotional and practical barriers to Pap test and increase participation in cervical screening despite some women’s concerns about test accuracy. Mailing to eligible women and assuring women about test accuracy could further optimize participation in screening.

**Electronic supplementary material:**

The online version of this article (doi:10.1186/s12885-015-1804-x) contains supplementary material, which is available to authorized users.

## Background

Increasing coverage by reaching women who are not screened or under-screened is essential for improving the effectiveness of cervical screening programs [1]. This is because most cancers in an organised screening program are diagnosed in women who have never been screened or are lapsed screeners [2]. Several strategies have been tried to improve screening participation, of which reminder letters have been shown to have a modest effect [3]. Nevertheless, barriers to having a Pap test remain.

Recently, it has been shown that human papillomavirus (HPV) testing as a primary screening test is more sensitive than a Pap test and provides better protection against cervical cancer [4]. Primary HPV testing also allows for self-sampling; a self-collected sample has been shown to have similar sensitivity (for underlying high grade cervical disease) to that of a practitioner-collected sample when a validated PCR based test is used [5].

Self-sampling for HPV testing has been shown to be more effective than a reminder letter to have a Pap test at improving cervical screening participation by women who are apparently never- and under-screened [6, 7]. However, information about women’s overall experience i.e. from the receipt of the self-sample pack at home to performing the test and mailing back the sample and receiving their results in the post is lacking. This information is essential for planning programs moving to primary HPV screening where self-sampling might be an option for women who do not attend cytological screening.

Prior to the start of a trial of home delivered HPV self-sample kits, we conducted four focus groups, including never- and under-screened women aged 30–69 years [8]. In the focus groups, women were positive towards the idea of self-sampling but expressed concerns about test accuracy and were not confident that the self-sampling would give the same results as a practitioner administered test [8]. However, women in the focus groups were only shown the device (dry flocked swab) and opinions were based on perceptions rather than experience. Few studies have reported on women’s actual experience with self-sampling, their preferences for cervical screening in the future, and the rationale behind these preferences especially among non-attendees of a screening program who have taken up an offer of self-sampling [9–12]. Only two studies have reported reasons for declining self-sampling among non-attendees of routine screening [9, 10].

This paper reports on a survey of never- and under-screened women who were randomised in a trial of self-sampling in Victoria, Australia, to document their experience with home-based HPV self-sampling and their views about self-sampling for cervical screening in future. Additionally, we investigated women’s reasons for not previously having had a Pap test, or an up-to-date one.

## Methods

Australia’s National Cervical Screening Program was introduced in 1991. Current policy recommends that sexually active women should be screened every 2 years with Pap tests, beginning at age 18 years (or 2 years after onset of sexual activity, whichever is later) until 69 years [13]. Pap testing is primarily provided through general practice and other primary healthcare settings with up to 85 % rebates available from Medicare (Australia’s publicly funded universal health care system) [14]. Eight jurisdictional cervical registries (Pap test registries) underpin the Program by (i) sending reminder letters, (ii) providing a safety net for follow-up of women with abnormal Pap smears, (iii) providing laboratories with screening histories to help with accurate reporting of tests and (iv) providing laboratories with quantitative data to assist with quality assurance [13]. The Victorian Cervical Cytology Registry (VCCR) is one of the jurisdictional registers and operates under the Victorian Cancer Act. The VCCR records details of almost all Pap tests performed in Victoria (<1 % opt-off). It also records details of hysterectomy, where these are provided by the woman or her practitioner. Participation in cervical screening in Victoria for 2 year (2012–2013) and 3 year (2011–2013) periods was 60.4 and 72.7 % respectively [2]. In 2017, Australia will be moving to five yearly primary HPV testing commencing at age 25, where self-sampling will be made available through medical or nurse practitioners (who will offer main stream cervical screening), in clinics, to women who are never- or under-screened, as per the revised policy, and who do not want to undergo a gynaecological examination [14].

**Fig. 1:**
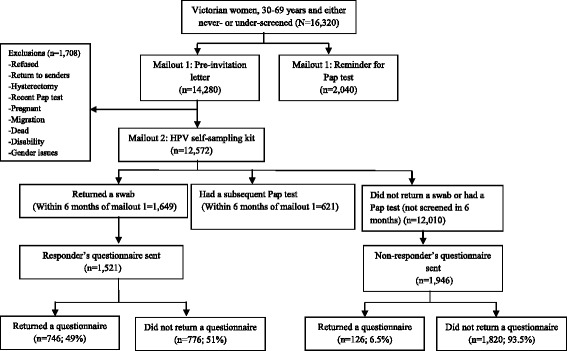
Participants in the iPap trial and questionnaires mailed and returned

The survey was conducted as a part of the iPap trial, a randomized controlled trial of home-based HPV self-sampling for improving participation in cervical screening by apparently never- and under-screened women [15]. Apparently never-screened women were women who were in the Victorian Electoral Roll and for whom no match was found in the VCCR indicating that no cervical screening episode had ever been recorded for these women (the two databases were matched on name, address and date of birth). Under-screened women were women whose last Pap test record in the VCCR was between 5 and 15 years ago. Women were eligible for the trial if they were 30–69 years, resided in Victoria, never-screened or under-screened, had not had a hysterectomy, and were not pregnant at the time of the study. No information on eligibility (other than age) was available for never-screened women prior to randomisation as by definition these women did not exist in the register that records details of screening history and hysterectomy. A total of 16,320 women were randomly allocated to either receive a) a pre-invitation letter followed a few weeks later by an HPV self-sampling kit (*n* = 14,280), or b) just a reminder letter to attend for a Pap test (*n* = 2040) in a 7:1 randomization ratio, and stratified by screening history (never- and under-screened). The self-sampling device was a nylon tipped flocked swab (Copan Italia, Brescia, Italy) enclosed in a dry plastic tube. A written and pictorial instruction sheet was enclosed detailing sample collection and postage. The mailout took place in 34 batches between March and July 2014. A kit was not mailed if the women opted out or reported ineligibility e.g. hysterectomy, recent Pap test, pregnancy, migration, death, disability, gender issues. A kit was also not mailed if the letter was returned to sender (Fig. 1). The primary outcome of the trial was return of a completed HPV self-sampling kit or the notification of a Pap test result to the VCCR measured at 3 and 6 months after the mailout of pre-invitation letter. The details of the trial design are described elsewhere [15].

A total of 1649 women returned a self-sample within 6 months of the mail out of the letters. Of these 1521 (92.2 %) were mailed a questionnaire after they were sent their results letters. We also sent questionnaires to 1946/ 12,010 women in the self-sampling arm (i.e. in the first seven of 34 batches) who did not return a self-sample or have a Pap test and whose letter did not come back return to sender or who did not report a hysterectomy, recent Pap test or pregnancy (Fig. 1). Time and resources allocated to the trial did not permit us to mail to all women in the self-sampling arm of the trial; especially the group that did not return a self-sample nor had a Pap test. We therefore used a sample of all participants and the most efficient way to achieve this was to use the women in the first batches of the trial. Given that the batches were randomly ordered, non-respondents in the first seven batches should be similar to those in the rest of the trial. The questionnaires were completed anonymously. The Human Research Ethics Committee of the Victorian Department of Health approved the study. Informed consent was waived for the main trial and the survey was conducted as a part of this trial. However, a cover letter explaining the purpose of the questionnaire and its anonymity was mailed to women in the survey.

Different questionnaires were designed for women who returned a self-sample and those who did not (Additional file 1). Those who returned a self-sample were asked about their experience with self-sampling, their willingness to participate in future self-sampling and their preference for collection at home or clinic. Women who did not return a self-sample were asked about reasons for not attending regular cervical screening, for not returning the kit, and what things would have helped them do so. Common to both questionnaires were questions on age, postcode of residence, screening history and reasons for never/not having had a recent Pap test. Age was reported in 10-year age categories (30–39, 40–49, 50–59 and 60+ years) and socioeconomic status (SES) as quintiles (1 being the lowest and 5 being the highest). SES is an area level variable assigned to women based on the 2011 Socio-economic Index of Areas (SEIFA), a composite measure of relative socio-economic disadvantage, as determined from their postcode of residence [16]. Screening history was classified as no history of having a Pap test, had a Pap test within last 5 years, ≥5 years since last Pap test or unsure of the time since last Pap test as self-reported by women. The questionnaires were completed anonymously and so could not be linked to the Victorian Pap test Register or any other data. We verified the self-reported Pap test done in Victoria for the main trial, which revealed that the reporting was reliable if the woman was never-screened (i.e. reported no Pap test) or if the last Pap test was in the recent past (data unpublished). However, it is possible that some may underestimate the time elapsed since their last Pap test, especially if the Pap test was in the distant past. We were also unable to verify self-reported Pap tests done interstate or overseas as this is beyond the scope of the current registers. Women were asked to give one main reason (single response) and up to three other reasons (multiple responses) for never/not having had a Pap test from a list of options, developed from previous research in Victoria on reasons for not screening (Additional file 1) [8, 17]. The main reason for not having a Pap test was also explored by self-reported screening status (i.e. never-screened, screened within 5 years and under-screened) in the group that returned both the self-sample and the questionnaire. We do not report the socio-demographic characteristics of women who did not return a self-sample in the iPap trial but returned a questionnaire given the very low response rate in this group (7 %). All the questions were close-ended questions. At the end of each questionnaire there was an option for open answer/comments where women were asked to make general comments about self-sampling (Additional file 1). Percentages were calculated for each answer and presented accordingly. Open answers or quotes are used to illustrate a survey finding where a need for better understanding was required. All data were analysed using Stata version 11.1 (StataCorp, College Station, TX).

## Results

### Response rate and socio-demographic characteristics

A total of 3468 questionnaires were mailed: 1521 to women who returned a self-sample as part of the iPap trial and 1946 to women who neither returned a self-sample nor had a subsequent Pap test. Questionnaires were returned by 746 (49 %) and 126 (7 %) women respectively.

Table 1 compares the socio-demographic characteristic of questionnaire responders and non-responders among the 1521 women who returned a self-sample and who were mailed a questionnaire. Questionnaire responders were more likely to be older (*p* = 0.002), from higher SES (*p* < 0.001) and screened within 5 years (*p* < 0.001). The median age (interquartile range) of women who did not return a self-sample but returned a questionnaire was 56 (44–64) years; 21 % reported they were never-screened, 39 % under-screened, 31 % screened within 5 years and 9 % unsure about screening history (data not shown).

**Table 1:** Demographic characteristic of questionnaire responders and non-responders among those that returned a self-sample and were mailed a questionnaire

	Women in the iPap trial who returned a self-sample					*p*-value
Characteristics	Questionnaire mailed^a^	Questionnaire responder		Questionnaire non-responder		
Age (years)	N	n	% row	n	% row	
30–39	429	177	41.3	252	58.7	0.002
40–49	350	175	50.0	175	50.0	
50–59	331	165	50.0	166	50.0	
60+	411	223	54.3	188	45.7	
SES^c^						
1 (lowest)	334	105	31.4	229	68.6	<0.001
2	286	119	41.6	167	58.4	
3	327	124	37.9	203	62.1	
4	301	188	62.5	113	37.5	
5 (highest)	273	199	72.9	74	27.1	
Screening history^b^						
No Pap test	409	163	40.0	246	60.0	<0.001
Yes, <5 years	256	229	89.5	27	10.5	
Yes, ≥5 years	809	344	42.5	465	57.5	
Unsure/Don’t know	47	5	10.6	42	89.4	

^a^Total women in the iPap trial who returned a self-sample and who were mailed a questionnaire (*n* = 1521)

^b^Self-reported screening history

^c^SES is an area level variable assigned to women based on the 2011 Socio-economic Index of Areas (SEIFA)

## Experience of self-sampling participants

### Reasons for not having a Pap test

Overall, 553 (74 %) women provided one main reason for not having a Pap test (Table 2), and the most common reason was embarrassment about having the test performed by a doctor (18 %). The most common reason that never-screened women had not had a Pap test was that they had never had sex (24 %); followed by believing that having the test performed by a doctor would be embarrassing (19 %). For the under-screened women, the most common reason was embarrassment at having a Pap test (16 %), followed by it being hard to find time (15 %).

**Table 2:** Main reason and other reasons for not having had a Pap test

Reasons	Returned a self-sample and a questionnaire (*n* = 746)^d^										Returned a questionnaire but not a self-sample (*n* = 126)^e^			
	Main reason (*n* = 553)^b^								Other reasons		Main reason^b^(*n* = 92)		Others reasons(*n* = 126)^c^	
	Overall		Never-screened		Screened <5 years		Under-screened							
	(*n* = 553)^a^		(*n* = 144)		(*n* = 122)		(*n* = 281)		(*n* = 746)^c^		ᅟ		ᅟ	
	n	%	n	%	n	%	n	%	n	%	n	%	n	%
1. I don’t think I need a Pap test	43	7.8	20	13.9	4	3.3	19	6.8	85	11.4	4	4.4	15	11.9
2. I don’t know if or when I should have a Pap test	19	3.4	10	6.9	7	5.7	2	0.7	57	7.6	2	2.2	4	3.2
3. I am not having sex	33	6.0	17	11.8	5	4.1	11	3.9	94	12.6	4	4.4	12	9.5
4. I have never had sex	39	7.1	35	24.3	1	0.8	3	1.1	25	3.4	11	11.9	2	1.6
5. I have had a hysterectomy	39	7.1	3	2.1	3	2.5	32	11.4	28	3.8	40	43.5	2	1.6
6. A Pap test from a doctor is embarrassing	97	17.5	28	19.4	23	18.9	45	16.0	167	22.4	5	5.4	16	12.7
7. A Pap test from a doctor is painful or uncomfortable	58	10.5	10	6.9	13	10.7	34	12.1	111	14.9	5	5.4	7	5.6
8. I have had a bad experience in the past having a Pap test	32	5.8	0	0	4	3.3	26	9.3	71	9.5	1	1.1	4	3.2
9. I don’t feel comfortable asking for a Pap test from my doctor	25	4.5	8	5.6	4	3.3	12	4.3	125	16.7	2	2.2	4	3.2
10. My doctor has not suggested a Pap test	21	3.8	4	2.8	2	1.6	15	5.3	97	13.0	1	1.1	13	10.3
11. It is hard to find the time to have a Pap test	75	13.6	3	2.1	30	24.6	42	14.9	93	12.5	9	9.8	8	6.4
12. It is hard to find the right doctor or get an appointment	33	6.0	2	1.4	11	9.0	20	7.1	102	13.7	3	3.3	4	3.2
13. It is hard to travel to an appointment	6	1.1	0	0	1	0.8	5	1.8	32	4.3	1	1.1	1	0.8
14. It is too expensive to have a Pap test	5	1.0	0	0	1	0.8	4	1.4	25	3.4	0	0	0	0
15. I have not received a reminder letter to have a Pap test	27	4.9	4	2.8	13	10.7	10	3.6	73	9.8	3	3.3	5	4.0
16. I don’t think Pap test results are accurate enough	1	0.2	0	0	0	0	1	0.4	15	2.0	1	1.1	1	0.8

^a^The overall includes never-screened (*n* = 144), under-screened (*n* = 281), screened in the last 5 years (*n* = 122) and unsure/don’t know (*n* = 6)

^b^Single response question where women were asked to give one main reason

^c^Multiple response question where women were asked to give up to three other reasons

^d^Of the 746 women who returned a self-sample and a questionnaire, 131 (17.5 %) did not provide a main reason and 62 (8.3 %) provided more than one main reason that was included with the other reasons as we were unable to determine the main reason

^e^Of the 126 women who did not return a self-sample but returned a questionnaire, 31 (24.6 %) did not provide a main reason and 3 (2.4 %) provided more than one main reason that was included with the other reasons as we were unable to determine the main reason

### Experience with HPV self-sampling

Of the 746 who did the self-sampling and returned a questionnaire, 737 (99 %) provided a response to at least one of the statements related to the experience (Table 3). The process of doing the test was rated very highly, with almost all women saying the instructions were very clear (98 %) and most finding the swab easy to use (95 %), especially those who had had a Pap test in the past (96 % versus 91 %). Women’s confidence in performing the test was also high, with 81 % very confident that they did it correctly, more so for the ever-screened (83 % versus 77 %). The test was also perceived to be very convenient (91 %). The majority also found self-sampling was not embarrassing (92 %) or painful (82 %). However, a quarter found it a little uncomfortable and 15 % reported it to be a little painful. When stratified by screening history, there were more never-screened than ever screened women who felt a little pain (19 % versus 14 %), were a little uncomfortable (35 % versus 22 %) and a little embarrassed doing the self-sample (12 % versus 4 %).

**Table 3:** Experience with self-sampling among those that returned a self-sample and a questionnaire

Experience with self-sampling (row %)	*n* = 746	Not at all		A little		Very much		Unsure	
		n	%	n	%	n	%	n	%
1. I thought the instructions were clear	737	4	0.5	10	1.4	721	98.0	2	0.3
Never-screened	162	2	1.2	1	0.6	158	97.5	1	0.6
Ever-screened	575	2	0.4	9	1.6	563	97.9	1	0.2
2. It was easy to use the swab	728	8	1.1	32	4.4	688	94.5	0	0
Never-screened	162	4	2.5	11	6.8	147	90.7	0	0
Ever-screened	566	4	0.7	21	3.7	541	95.5	0	0
3. Taking the sample with the swab was painful	715	585	81.8	108	15.1	19	2.7	3	0.4
Never-screened	159	122	76.7	30	18.9	5	3.1	2	1.3
Ever-screened	556	463	83.3	78	14.0	14	2.5	1	0.2
4. Taking the sample with the swab was uncomfortable to do	714	514	72.0	180	25.2	20	2.8	0	0
Never-screened	158	99	62.7	56	35.4	3	1.9	0	0
Ever-screened	556	415	74.6	124	22.3	17	3.1	0	0
5. I felt embarrassed	708	650	91.8	39	5.5	17	2.4	2	0.3
Never-screened	155	130	83.9	19	12.3	5	3.2	1	0.7
Ever-screened	553	520	94.0	20	3.6	12	2.2	1	0.2
6. It was convenient	729	48	6.6	17	2.3	664	91.1	0	0
Never-screened	162	11	6.8	4	2.5	147	90.7	0	0
Ever-screened	567	37	6.5	13	2.3	517	91.2	0	0
7. I am confident I did it correctly	728	29	3.9	81	11.1	592	81.3	26	3.4
Never-screened	161	7	4.4	24	14.9	124	77.0	6	3.7
Ever-screened	567	22	3.9	57	10.1	468	82.5	20	3.5

The following were typical comments:

*“Having not had a Pap test before, I was grateful for the kit being sent out. It wasn’t invasive and put my mind at ease having good results.”*

*“What a fabulous development – quick, easy, free, non-embarrassing and no appointment required. Results posted to one’s house. Hope this continues and more medical tests like this are developed in the future.”*

When the 573 women who had previously had a Pap test were asked to compare that experience with self-sampling, most women found self-sampling easier (93 %), more convenient (98 %), less embarrassing (94 %) and less uncomfortable (90 %). However, when asked about accuracy, 57 % were unsure which was better and another 20 % thought there was no difference between the two methods (Table 4).

**Table 4:** Comparing self-sample taken at home to the last Pap test performed by a doctor

Comparison	*n* = 573	Self-sample		Pap test		No difference		Unsure	
		n	%	n	%	n	%	n	%
Easier	525	490	93.3	11	2.1	20	3.8	4	0.8
More convenient	519	507	97.7	3	0.6	4	0.8	5	1.0
Less embarrassing	507	478	94.3	2	0.4	24	4.7	3	0.6
Less uncomfortable	494	446	90.3	12	2.4	34	6.9	2	0.4
More accurate	469	67	14.3	40	8.5	93	19.8	269	57.4

The following were typical comments:

*“This was very quick and easy to do. Much better than having to make an appointment then wait in a waiting room for up to an hour to see a doctor for the simple task.”*

*“The convenience, efficient use of time, privacy and comfort of the self-sampling has got me interested again in having a regular pap test.”*

*“Got positive result from self sample, had to go to a doctor, recheck, she got normal results & charged me”*

### Preference for self-sampling in future

Women who did self-sampling were asked if they would prefer to see a health professional or take their own sample for cervical screening in future: 734 out of 746 (98 %) reported a preference; of those, 88 % (*n* = 644) preferred to take their own sample at home, 1.2 % (*n* = 9) preferred to take their sample at a medical clinic, 6 % (*n* = 42) preferred a health professional to take their sample and 4 % (*n* = 27) were not sure. A few women also reported that they did not intend to screen again (*n* = 12, 1.6 %). Of those that preferred to take their own sample (*n* = 653), either at home or a clinic, the top two reasons for their preference was that the self-sample test was simple to do (59 %) and did not require an appointment with a doctor (55 %).

The following were typical comments:

*“I felt very comfortable with the self sample test. And would like to do my own test from home from now on”*

*“loved it, would do it regularly if I can do it myself. Best idea ever!!! and it was free!*

*“…I would even pay more than a doctor’s visit if it was an option to purchase these kits”*

## Non-participants to the offer of self-sampling

### Reasons for not-returning a self-sample and intention to screen

We asked women who did not return a self-sample or who did not have a subsequent Pap test what they did with the kit (Table 5). Of the 126 who did not screen but returned a questionnaire, 111 (88 %) remembered receiving the kit in the mail. Of the 111 women, 16 (14 %) had not opened the kit, 66 (60 %) had opened the kit but did not self-sample, another 16 (14 %) had opened it and threw it away and 8 (7 %) threw it away unopened, 3 (3 %) completed it but not returned it and 2 (2 %) had completed and returned it.

**Table 5:** Intention to complete the test if women have not returned the self-sample

What have you done with the kit?	Total		Intention to complete and return the kit		
	(*n* = 111)				
	n	%	No (*n* = 78)	Unsure (*n* = 14)	Yes (*n* = 19)
I have opened it but not done it	66	59.5	44 (66.7)	10 (15.2)	12 (18.2)
I haven’t opened it	16	14.4	10 (62.5)	4 (25.0)	2 (12.5)
I have opened it, but threw it away	16	14.4	16 (100)	0	0
I threw it away unopened	8	7.2	8 (100)	0	0
I have completed it but not returned it	3	2.7	0	0	3 (100)
I have completed and returned it	2	1.8	0	0	2 (100)

We also asked women about their intention to complete and return the kit. Of the 111 women, 70 % (*n* = 78) reported that they had no intention to complete and return the kit, 17 % (*n* = 19) still intended to complete and return the kit and another 13 % (*n* = 14) were unsure. The main reasons for not doing the self-sample test were that women thought they did not need it because they have had a hysterectomy (42 %), 17 % believed that only health professionals should do this sort of test, 10 % were not sexually active, and 6 % did not think it is as reliable as a Pap test. Hysterectomy (44 %) and never having had sex (12 %) were also the two main reasons, reported by women who did not return the self-sample, for not attending regular screening (Table 2). Of those who still intend to complete and return the test, the two main reasons for not having done it yet were that 63 % forgot about the kit or had not got around to it, 21 % were too busy and another 10 % thought it looked like it could be painful/uncomfortable and 5 % were worried about the test results.

Women who were unsure (*n* = 14) were also asked to report two things that would enable them to make a decision regarding whether to do the test or not. Talking to their doctor (*n* = 7) or getting more information on the test (*n* = 5) or being sure that it was as reliable as a Pap test (*n* = 3) were some of the things that women reported would help them make a decision regarding doing the test. Other suggestions to enable decision making, were seeing a demonstration video in the website (*n* = 1) or talking to someone who has done the test (*n* = 1).

The following were typical comments:

*“No, I prefer to get advice by a doctor who has experience about pap testing, that's why I didn't complete the self kit Pap test sample.”*

*“I also felt I wasn’t qualified to do this and wasn’t sure if able to do it correctly.”*

*“It reminded me to ask my new doctor for a pap test. Thanks! I think it is a good idea for those who don’t have good access to a doctor, or who can’t afford to have a pap test.”*

## Discussion

### Key findings

Overall, common reasons for not having a Pap test were related to embarrassment, not having time, or that a Pap test was painful and uncomfortable. Participants found the home-based self-sampling less embarrassing and less uncomfortable compared with their last Pap test experience. Women who self-sampled thought the instructions were clear and were generally confident that they did the test correctly; however, many were unsure about the test accuracy. The majority preferred to take their own sample at home if offered in future because it was simple and did not require an appointment with a doctor.

### Reasons for not participating in regular screening

The main reason for not participating in regular screening provided by women who participated in iPap trial was emotional/attitudinal i.e. Pap test from a doctor is embarrassing (18 %), with the second most common reason being related to practical issues such as finding time to do a Pap test (14 %). This was similar to the findings in the UK trial for non-attendees where emotional barriers (uncomfortable/painful/unpleasant etc.) were more common than practical barriers (lack of time/busy/no childcare etc.) [18] but unlike that in a Dutch study where the main reason for non-attendance was that women forgot to schedule an appointment (32.3 %) [10]. In an Italian study, the main reason for failing to comply with a previous screening invitation was a recent Pap smear done outside the screening program (40.6 %) with the second most common reason being related to practical issues such as no time (23 %) [11]. In another population based survey in England, practical barriers were more predictive of screening uptake than emotional barriers [19]. No previous studies have reported barriers by screening status (never- and under-screened). In our survey, the main reason provided by never-screened women, who participated in self-sampling, for not having a Pap test was that they had never had sex. In Australia, only women who have ever been sexually active are actively encouraged to participate in screening.

### Self-sampling can overcome main barriers to cervical screening

Women found self-sampling more convenient, less embarrassing, less uncomfortable and less painful than their last Pap test. This is encouraging as self-sampling is likely to overcome the two main barriers reported by women in this study (related to test and time). The experience was similar for never- and ever-screened except that more never-screened women reported a little more pain, discomfort and embarrassment than ever-screeners. Nevertheless, 88 % of the self-sampling participants preferred to take their own sample at home in future and this was because it was simple to do and did not require a doctor’s appointment. Similar reasons were also reported in other studies that included non-attendees of regular screening who took up self-sampling [9–12, 20].

### Improving participation using self-sampling

One of the issues regarding self-sampling identified in our focus group and in other studies was concern about test accuracy [8]. While the majority of women who did the self-sampling in our trial reported that they were confident about doing it correctly (81 %), only 14 % thought it was more accurate than a Pap test performed by a doctor. These findings about confidence are in line with the results of a large trial among non-attendees in The Netherlands (*n* = 30,130) which compared a lavage with a brush self-sampling device. In this study, 20 % of all participants reported that they were concerned about taking the self-sample correctly with no difference between the two groups [12]. In another study among non-attendees of a cervical screening program who participated in self-sampling in Finland, around 88 % felt confident that they collected the sample successfully using a lavage-like device and a similar proportion (83 %) trusted the test results [9]. It is difficult to pinpoint if the difference between our study and the study in Finland related to trust in the self-sampling test is due to the different devices used in the two studies, the way the information was communicated, or the extent of details covered. Moreover, in the Finland study, around 22 % of the participants also reported that they felt insecure during the sample taking and commonly reported concerns related to the plunger of the device not releasing properly, fluid leaking out during sample taking and the small volume of the sample collected using device indicating that a certain level of doubt will remain [9]. Furthermore, 13 % (14/111) of women who did not do the self-sampling but returned a questionnaire reported they were unsure about their intention to do the test. These women said that talking to a doctor, or getting more information on the test and being sure that the home-based test was as reliable as a Pap test would assist their decision-making. Another 20 % (19/111) who did not return the self-sample still intended to do the test and the questionnaire acted as a reminder for them. This is an indication that there will be some women who will not prioritize screening even after being mailed a kit and a further reminder might trigger their participation; this was evident in a trial of home-based self-sampling in Sweden where high participation (39 %) was achieved for under-screened women when a reminder letter was sent if the kit was not returned in time [20].

The very low questionnaire response rate (7 %) by women who did not return a self-sample limits the validity of the results of our study for this group. These women were non-responders to participation in the main trial so a low rate of response to the survey was not unexpected. These women mostly used the questionnaire to report their hysterectomy status, which was also the main reason for not screening previously. This was different from findings in the Finnish study where a recent Pap test elsewhere was the main reason provided by the majority (70 %) who did not take part in the self-sampling but returned a questionnaire (10 % response rate) [9] reflecting differences in the target population to our study. In another study in The Netherlands, only 2.3 % of those who did not self-sample but returned a questionnaire reported that they preferred an invitation for regular screening [10]. In our study, no information on eligibility was available for never-screened women prior to randomisation given that these women did not have records in the register regarding details of prior Pap tests or hysterectomy status. However, when self-sampling is possibly made available in the revised National Cervical Screening Program of Australia (with a move to primary HPV testing in May 2017), through medical or nurse practitioners, in clinics, the issue of eligibility may be resolved through direct conversation with the woman about her previous medical history and reasons for not screening previously or delaying screening. With home-based self-sampling, the issue of eligibility needs to be clearly communicated in the materials sent with the kit.

### Strengths and limitations

Resource and time constraints did not permit us to mail to all women in the self-sampling arm of the trial for feedback about their experience. Of those mailed, the response to questionnaires by those who screened using self-sampling was modest and those who were long term non-attenders and from lower socioeconomic backgrounds are underrepresented. This is one of the few studies to report on self-sampling experience of never- and ever-screened women who did not attend regular screening but who took up the offer of self-sampling and key areas to focus on to optimise this as a strategy for improving coverage. A major limitation of our study is the very low response rate of the women who did not return a self-sample. Non-responders are a hard group to reach in any screening program and little is known about their reasons for non-participation and things that would encourage them to participate. Although a small and potentially biased group, the study provides some insight into their information needs and what would enable them to participate.

## Conclusion

In conclusion, home based self-sampling can overcome emotional/attitudinal and practical barriers to Pap testing and increase participation in cervical screening because women view it as less embarrassing, less uncomfortable, more convenient and easier than having a Pap test. These findings are encouraging considering women would not have heard of self-sampling testing for HPV outside this trial. Although many women were unsure about the test accuracy, they still preferred to self-sample in future as it was simple and did not require an appointment. Among those that did not perform the self-sample, some were unsure and decision-making was dependent on getting more information or being sure that the test was reliable. Therefore to optimize this intervention as a strategy and to improve participation, provision of clear information and education around test accuracy of self-sampling, as well as clear instructions about how to take the sample, to both the providers and their clients will be very important. Information on eligibility will also have to be clearly communicated if kits are to be mailed home for self-sampling.

## Additional file

Additional file 1:
**HPV Self-Sampling Survey.** (ZIP 115 kb)

## Abbreviations

SES: Socioeconomic status
SEIFA: Socio-economic Index of Areas
HPV: Human papillomavirus
PCR: Polymerase chain reaction
