# Supplementary material for: Women’s experience with home-based self-sampling for human papillomavirus testing
Source: BMC Cancer. 2015 Nov 4;15:849. doi: 10.1186/s12885-015-1804-x (PMC4634718; doi:10.1186/s12885-015-1804-x)
Supplement: Additional file 1: — HPV Self-Sampling Survey. (ZIP 115 kb) [file 12885_2015_1804_MOESM1_ESM.zip › Additional file 1/7_Appendix 1_questionnaire for non-responders to self-samplingR2.doc]

**HPV Self-Sampling Survey**

1. **What is your age (in years)?**	___________
2. **What is your current residential postcode?**	___________
3. **Have you ever had a Pap test done by a health professional?**

|  | 1. Yes | ☐ |
| --- | --- | --- |
|  | 2. No | ☐ |
|  | 3. Unsure | ☐ |
| **If yes, how long ago was your last Pap test?** | | |
|  | 1. Within the last 5 years | ☐ |
|  | 2. 5 to 10 years ago | ☐ |
|  | 3. More than 10 years ago | ☐ |

4. **What are your main reasons for never having had a Pap test, or for not having had a recent Pap test? *Please select one main reason, then up to 3 other reasons that apply.***

|  | **Main reason**  **(select one only)** | **Other reasons**  **(select up to 3)** |
| --- | --- | --- |
| 1. I don’t think I need a Pap test | ☐ | ☐ |
| 2. I don’t know if or when I should have a Pap test | ☐ | ☐ |
| 3. I am not having sex | ☐ | ☐ |
| 4. I have never had sex | ☐ | ☐ |
| 5. I have had a hysterectomy | ☐ | ☐ |
| 6. A Pap test from a doctor is/would be embarrassing | ☐ | ☐ |
| 7. A Pap test from a doctor is/would be too painful or uncomfortable | ☐ | ☐ |
| 8. I have had a bad experience in the past having a Pap test | ☐ | ☐ |
| 9. I don’t/wouldn’t feel comfortable asking for a Pap test from my doctor | ☐ | ☐ |
| 10. My doctor has not suggested a Pap test | ☐ | ☐ |
| 11. It is hard to find the time to have a Pap test | ☐ | ☐ |
| 12. It is hard to find the right doctor or get an appointment | ☐ | ☐ |
| 13. It is hard to travel to an appointment | ☐ | ☐ |
| 14. It is too expensive to have a Pap test | ☐ | ☐ |
| 15. I have not received a reminder letter to have a Pap test | ☐ | ☐ |
| 16. I don’t think Pap test results are accurate enough | ☐ | ☐ |

5. **Do you remember receiving the “self-sample kit” in the mail?**

*(the kit contained instructions, a swab and a reply paid envelope)*

1. Yes	☐ →	*Please continue with the survey*
2. No	☐ →	*You have now completed the survey. Thank you.*
6. **What have you done with the kit?**
1. I have opened it but not done it	☐ →	*Please continue with the survey*
2. I haven’t opened it	☐ →	*Please continue with the survey*
3. I have opened it, but threw it away	☐ →	*Please answer “No” in Question 7 and indicate why*
4. I threw it away unopened	☐ →	*Please answer “No” in Question 7 and indicate why*
5. I have completed it but not returned it	☐ →	*Please go to Question 8*
7. **Do you plan to complete and return the kit?**

| ☐ **Yes** | **Why haven’t you completed the kit yet?** *Please select up to two main reasons* | | | |
| --- | --- | --- | --- | --- |
|  | 1. I am too busy | | ☐ | |
|  | 2. I forgot about it/haven’t got around to it | | ☐ | |
|  | 3. It seems complicated | | ☐ | |
|  | 4. It looks like it could be painful/uncomfortable | | ☐ | |
|  | 5. I am worried about the result | | ☐ | |
|  | 6. Other reason (please specify) | | | |
|  |  | |  | |
| **☐ Unsure** | **What things would help you decide whether to do the test?** *Please select up to two main reasons* | | | |
|  | 7. Talking to someone who has done it | | ☐ | |
|  | 8. Being sure it was as reliable as a Pap test | | ☐ | |
|  | 9. Getting more information | | ☐ | |
|  | 10. Talking to my doctor | | ☐ | |
|  | 11. Other reason (please specify) | | | |
|  |  | |  | |
| **☐ No** | **Why did you decide not to do the kit? Please select one main reason, then up to 3 other reasons** | | | |
|  |  | **Main reason**  **(select one)** | | **Other reasons**  **(up to 3 reasons)** |
|  | 12. I just wasn’t interested | ☐ | | ☐ |
|  | 13. I was too embarrassed or uncomfortable | ☐ | | ☐ |
|  | 14. I didn’t know what it was for | ☐ | | ☐ |
|  | 15. I don’t need it because I have had a hysterectomy | ☐ | | ☐ |
|  | 16. I don’t need it because I am not sexually active now | ☐ | | ☐ |
|  | 17. I don’t need it because I have never been sexually active | ☐ | | ☐ |
|  | 18. I am not at risk of cervical cancer | ☐ | | ☐ |
|  | 19. I believe that only a health professional should do this sort of test | ☐ | | ☐ |
|  | 20. I don’t think it would be as reliable as a Pap test | ☐ | | ☐ |
|  | 21. Treatment for cancer is worse than the disease | ☐ | | ☐ |
|  | 22. I believe cancer can’t be treated effectively anyway | ☐ | | ☐ |
|  | 23. Other reason (please specify) | | | |
|  |  | | | |

8. **Are there any comments about the self-sampling test that you would like to make?**

**Thank you for your feedback!**

**Please return this survey form in the enclosed Reply Paid envelope,**

**or post to PO Box 310, East Melbourne, VIC 8002.**
