# Supplementary material for: Women’s experience with home-based self-sampling for human papillomavirus testing
Source: BMC Cancer. 2015 Nov 4;15:849. doi: 10.1186/s12885-015-1804-x (PMC4634718; doi:10.1186/s12885-015-1804-x)
Supplement: Additional file 1: — HPV Self-Sampling Survey. (ZIP 115 kb) [file 12885_2015_1804_MOESM1_ESM.zip › Additional file 1/8_Appendix 1_questionnaire for responders to self-samplingR2.doc]

Dear Participant,

We recently sent you a self-sampling kit to test for Human Papillomavirus (HPV). Thank you very much for returning your kit.

Please find enclosed a brief survey about HPV self-sampling. We would be very grateful if you would give us some feedback about your experience using the kit, as this will help us improve the process for other women. A reply paid envelope is enclosed for your convenience.

The survey is anonymous, and your responses will not be linked back to you.

If you are able to complete and return the survey, this would be very helpful and much appreciated.

Yours sincerely

Associate Professor Dorota Gertig

Medical Director

1. **What is your age (in years)?**	___________
2. **What is your current residential postcode?**	___________
3. **Have you ever had a Pap test done by a health professional?**

| 1. Yes | ☐ |
| --- | --- |
| 2. No | ☐ |
| 3. Unsure | ☐ |

a. ***If yes, how long ago was your last Pap test?***

| 1. Within the last 5 years | ☐ |
| --- | --- |
| 2. 5 to 10 years ago | ☐ |
| 3. More than 10 years ago | ☐ |

4. **What are your main reasons for never having had a Pap test, or for not having had a recent Pap test?** *Please select one main reason, then up to 3 other reasons that apply.*

|  | **Main reason**  **(select only 1)** | **Other reasons**  **(select up to 3)** |
| --- | --- | --- |
| 4. I don’t think I need a Pap test | ☐ | ☐ |
| 5. I don’t know if or when I should have a Pap test | ☐ | ☐ |
| 6. I am not having sex | ☐ | ☐ |
| 7. I have never had sex | ☐ | ☐ |
| 8. I have had a hysterectomy | ☐ | ☐ |
| 9. A Pap test from a doctor is/would be embarrassing | ☐ | ☐ |
| 10. A Pap test from a doctor is/would be too painful or uncomfortable | ☐ | ☐ |
| 11. I have had a bad experience in the past having a Pap test | ☐ | ☐ |
| 12. I don’t/wouldn’t feel comfortable asking for a Pap test from my doctor | ☐ | ☐ |
| 13. My doctor has not suggested a Pap test | ☐ | ☐ |
| 14. It is hard to find the time to have a Pap test | ☐ | ☐ |
| 15. It is hard to find the right doctor or get an appointment | ☐ | ☐ |
| 16. It is hard to travel to an appointment | ☐ | ☐ |
| 17. It is too expensive to have a Pap test | ☐ | ☐ |
| 18. I have not received a reminder letter to have a Pap test | ☐ | ☐ |
| 19. I don’t think Pap test results are accurate enough | ☐ | ☐ |

5. **Thinking about your experience of using the “self-sample kit” we posted you, please rate to what extent the following statements apply to you:**

|  | **Not at all** | **A little** | **Very much** | **Unsure/can’t say** |
| --- | --- | --- | --- | --- |
| 1. I thought the instructions were clear | ☐ | ☐ | ☐ | ☐ |
| 2. It was easy to use the swab | ☐ | ☐ | ☐ | ☐ |
| 3. Taking the sample with the swab was painful | ☐ | ☐ | ☐ | ☐ |
| 4. Taking the sample with the swab was uncomfortable to do | ☐ | ☐ | ☐ | ☐ |
| 5. I felt embarrassed | ☐ | ☐ | ☐ | ☐ |
| 6. It was convenient | ☐ | ☐ | ☐ | ☐ |
| 7. I am confident I did it correctly | ☐ | ☐ | ☐ | ☐ |

6. **In the future, would you prefer to see a health professional or take your own sample for cervical screening?**

|  | **Select only 1 reason** |
| --- | --- |
| 1. I would prefer a health professional | ☐ |
| 2. I would prefer to take my own sample at home | ☐ |
| 3. I would prefer to take my own sample at a medical clinic | ☐ |
| 4. I don’t intend to screen again | ☐ |
| 5. Don’t know/can’t say | ☐ |

a. **If you would prefer to take your own sample, please indicate your top 2 reasons for this:**

|  | **Top 2 reasons** |
| --- | --- |
| 1. The test is free | ☐ |
| 2. The test did not require an appointment with a doctor | ☐ |
| 3. The test is accurate | ☐ |
| 4. The test is less embarrassing | ☐ |
| 5. The test is simple to do | ☐ |
| 6. The test does not require the use of instruments (eg. speculum) | ☐ |

**Only answer the following question if you have had a Pap test in the past:**

7. **Thinking back to both your last Pap test performed by a doctor and the self-sample you took at home, which of the methods was:**

|  | **Self-sample** | **Pap test** | **No difference between the 2 methods** | **Unsure/can’t say** |
| --- | --- | --- | --- | --- |
| 1. Easier | ☐ | ☐ | ☐ | ☐ |
| 2. More convenient | ☐ | ☐ | ☐ | ☐ |
| 3. Less embarrassing | ☐ | ☐ | ☐ | ☐ |
| 4. Less uncomfortable | ☐ | ☐ | ☐ | ☐ |
| 5. More accurate | ☐ | ☐ | ☐ | ☐ |

8. **Are there any comments about the self-sampling test that you would like to make?**

**Thank you for your feedback!**

**Please return this survey form in the enclosed Reply Paid envelope, or post to**

**PO Box 310, East Melbourne, VIC 8002.**
